# A Radar-Based Smart Sensor for Unobtrusive Elderly Monitoring in Ambient Assisted Living Applications

**DOI:** 10.3390/bios7040055

**Published:** 2017-11-24

**Authors:** Giovanni Diraco, Alessandro Leone, Pietro Siciliano

**Affiliations:** National Research Council of Italy, Institute for Microelectronics and Microsystems, 73100 Lecce, Italy; alessandro.leone@le.imm.cnr.it (A.L.); pietro.siciliano@le.imm.cnr.it (P.S.)

**Keywords:** fall detection, vital signs monitoring, heart rate, respiration rate, ultra-wideband radar, micro-Doppler, supervised, unsupervised

## Abstract

Continuous in-home monitoring of older adults living alone aims to improve their quality of life and independence, by detecting early signs of illness and functional decline or emergency conditions. To meet requirements for technology acceptance by seniors (unobtrusiveness, non-intrusiveness, and privacy-preservation), this study presents and discusses a new smart sensor system for the detection of abnormalities during daily activities, based on ultra-wideband radar providing rich, not privacy-sensitive, information useful for sensing both cardiorespiratory and body movements, regardless of ambient lighting conditions and physical obstructions (through-wall sensing). The radar sensing is a very promising technology, enabling the measurement of vital signs and body movements at a distance, and thus meeting both requirements of unobtrusiveness and accuracy. In particular, impulse-radio ultra-wideband radar has attracted considerable attention in recent years thanks to many properties that make it useful for assisted living purposes. The proposed sensing system, evaluated in meaningful assisted living scenarios by involving 30 participants, exhibited the ability to detect vital signs, to discriminate among dangerous situations and activities of daily living, and to accommodate individual physical characteristics and habits. The reported results show that vital signs can be detected also while carrying out daily activities or after a fall event (post-fall phase), with accuracy varying according to the level of movements, reaching up to 95% and 91% in detecting respiration and heart rates, respectively. Similarly, good results were achieved in fall detection by using the micro-motion signature and unsupervised learning, with sensitivity and specificity greater than 97% and 90%, respectively.

## 1. Introduction

The population aged 65 and over, which is the fastest growing sector in developed countries [1], suffers from the highest morbidity and mortality rates due to age-related disorders (e.g., illness and functional decline) [2] and injury-related conditions (e.g., trauma and fractures) [3,4]. In this context, it is paramount to monitor older adults in their own homes, but it becomes challenging when family members or caregivers cannot be always available. Consequently, during the last years, the demand for unobtrusive sensing of human activities and behaviors as well as physiological parameters has increased notably in the ambient assisted living (AAL) domain. Indeed, automated sensor systems can help by continuously monitoring elderly for detection of dangerous situations and even for early prediction of health disorders, in order to provide timely medical assistance and alerts to caregivers.

Most of the current elderly monitoring systems are aimed to monitor activities [5] and vital signs [6] of elderly in their daily life for the automated detection of abnormal events, among which falls are without doubt one of the major healthcare concerns [7]. In fact, as some studies pointed out [8,9,10], the so called long-lie after a fall (more than one hour) increases risk of both hospitalization and death. Automatic fall-detection systems, saving time for the arrival of medical assistance, have the potential to reduce risk of these adverse health consequences [11]. On the other hand, respiration rate (RR) and heart rate (HR) are fundamental vital signs whose alterations may be correlated, especially during ageing, with the progress of physical illnesses (e.g., sleep-disordered breathing [12], congestive heart failure [13], and subclinical inflammation [14]) as well as mental and neuro-degenerative diseases (e.g., major depressive disorder [15]) and Parkinson disease [16,17]). Furthermore, the monitoring of vital signs can provide additional information useful for assessing the fall risk [18].

Although a variety of sensing technologies are available for continuous monitoring of activities and vital signs (as better discussed in Section 2), the emphasis on unobtrusiveness is especially important. In fact, it has been found by Wild et al. [19] that older adults are more likely to accept in-home sensing technologies when they are unobtrusive, i.e., they do not require wearing any device, do not interfere with daily life, do not require learning new technical skills and, above all, they do not capture video images. Indeed, it is only by means of a good acceptability that it is possible to provide a continuous monitoring, essential to produce long-term health data from which informative patterns can be extracted.

The ultra-wideband (UWB) radar is a promising sensing technology which fulfil such unobtrusiveness requirements, besides being privacy-preserving and able to detect both body and cardiorespiratory movements. In addition, UWB impulse radio (UWB-IR) technology offers additional advantages over continuous wave one (UWB-CW) which is up to now the most investigated in health-related monitoring applications. The UWB-IR is capable of operating over a larger bandwidth and wider range of frequencies than UWB-CW [20], which means submillimeter range resolution and high penetration power, enabling the detection of very small moving targets (e.g., vital signs) even through obstacles. The shorter pulse duration, lower than the total travel time of the wave even in case of multiple reflections, allows to effectively deal with multipath effects particularly insidious in indoor environments. The very low power spectral density (lower than −41.3 dBm/MHz [21]) prevents interferences with other radio systems operating in the same frequency range, which is an important requirement in AAL applications.

Along with sensing technology, another important aspect of a monitoring system is the detection methodology. This is especially critical for the detection of adverse events, such as falls, in which sensor data are not available due to the nature of the event being detected. In fact, a fall event affects negatively the health of the fallen person and its occurrence is quite rare [11]. Thus, ethical and practical reasons prevent the collection of sensor data regarding falls. Additionally, since each person falls in a different way, fall data from another person would not be useful, even if they were available in public datasets. Motivated by such considerations, a new research direction has emerged in recent years with the perspective of treating a fall as an abnormal activity, and thus reflecting real-life conditions where falls happen infrequently [22]. Under this perspective, fall detection algorithms are trained, calibrated and tuned by using sensor data collected from normal activities of daily living (ADLs), instead of using simulated falls.

To the best of the authors’ knowledge, no published study has investigated the usage of UWB-IR radar sensors for detecting both vital signs and abnormal activities (i.e., falls), in AAL contexts.

This paper is organized as follows. Section 2 reviews the related work. Section 3, firstly, provides an overview of the smart sensor system (SSS) presented in this study, then details the computational framework (i.e., the stages devoted to pre-processing, body movements and vital signs), and finally describes the experimental setup and validation procedure. Section 4 presents the research findings, which are discussed in Section 5. Some conclusions and remarks are given in Section 6.

## 2. Related Work

As mentioned in the Introduction, in-home monitoring solutions should be evaluated not only based on their detection performance (usually expressed in terms of accuracy, sensitivity and specificity), but also based on their acceptability by end-users. Under this perspective, the main issues of such systems can be traced to the adopted sensing technology and detection methodology, which will be used as guidance, in this section, to review the most relevant works.

Referring to the sensing technology, existing solutions can be roughly categorized based on the positioning modality of their sensing elements in two main categories [23]: wearable solutions and ambient (non-wearable) solutions. The former solutions require the subject to be tethered to a body-worn or closely located measurement device, resulting uncomfortable and unpractical for continuous monitoring in assisted living scenarios. However, slightly more comfortable wearable solutions involve the use of sensors embedded in garments and accessories (e.g., t-shirts, smartwatches, etc.), as better described below. The ambient solutions, instead, are based on sensor elements embedded or installed into the home environment, ranging from information-poor but well accepted devices (such as simple on/off switches, pressure sensors, infrared sensors, etc.) to more information-rich detectors, such as video cameras, but that raise privacy concerns.

On the methodological side, the most popular methodology used for detecting adverse events relies on supervised classifiers, which are trained with sensor data obtained by simulating both adverse and normal activities, with the involvement of healthy volunteers in laboratory controlled conditions. However, particularly in the case where adverse events were falls, some studies pointed out that detection algorithms trained with simulated falls exhibit poor performance when tested with older adults in real-world conditions [24,25]. Such considerations, together with the lack of fall data, motivate the use of unsupervised methods, able to autonomously learn the normal behavior of the monitored person when performing his/her ADLs [22]. After a sufficiently long observation period (behavior modeling), a fall event can be detected as an abnormal deviation from the modeled behavior.

The remaining of this section is organized in four parts. The section begins (Section 2.1) with an overview of wearable-based monitoring solutions, focusing mainly on the measurements of vital signs. The related works concerning ambient solutions are presented in Section 2.2. Here, the focus is on activity monitoring and fall detection. The last two Sections (Section 2.3 and Section 2.4), instead, are devoted to radar-based monitoring for detecting falls and vital sings, respectively.

### 2.1. Wearable Solutions

Regarding the measurement of vital parameters, and more specifically the heart activity, the golden standard is the electrocardiograph (ECG) [26], which involves various kind of electrodes (i.e., conventional Ag–AgCl suction, adhesive gel, etc.) attached to the skin on the chest and limbs. In regards to the respiration activity, the standard measurement technique is the transthoracic impedance plethysmography (IP), requiring skin electrodes placed on the chest, of which at least two must be ECG electrodes [27].

Focusing on the measurement of basic parameters, such as respiration-rate (RR) and heart-rate (HR), slightly more comfortable approaches may involve the use of textile dry or capacitive ECG electrodes, elastic bands around abdomen and/or chest (e.g., respiratory inductance plethysmography), optoelectronic sensors (e.g., Photoplethysmography (PPG)), and even pressure or accelerometer sensors. El-Amrawy and Nounou [28] evaluated the accuracy and precision of the most recent commercial, fitness wrist-wearable trackers (such as, Apple Watch, Samsung Gear Fit, Garmin Vivofit, and FitBit Flex—just to name a few), in measuring HR and step counts. They found that the evaluated trackers are relatively accurate (i.e., accuracy from 99.9% to 92.8%, and precision from 5.9% to 20.6%) and beneficial to estimate physical activity, e.g., travelled distance, calories burned, sleep monitoring.

Referring more specifically to health applications, several wearable medical devices have been demonstrated, as presented by Khan et al. in their pivotal review study [29], for measuring various vital sings, HR and RR as well as temperature, blood pressure, pulse oxygenation, and blood glucose. Such devices can be located in different body parts (e.g., arm, wrist, chest, and leg) depending on biosignal being measured, and make use of flexible/stretchable sensors and low-power silicon-based electronics. Moreover, wearable systems are adopted for monitoring not only health but also activities, mainly with the aim to provide timely help in case of emergency situations. In this case, the wearable device can be a simple manually-operated Personal Emergency Response System (PERS), not useful however in the case of loss of consciousness, or an automatic system equipped with motion sensors such as accelerometers, gyroscopes, and compasses [30].

Wearable solutions have the advantage of being usable “on the move” and showing relatively good detection performance, but their common drawbacks are the limited battery life [31], the need for on-board processing and/or wireless communication (both energy-demanding functions) [32], the inconvenience of having to remember to wear a device and the discomfort caused by the device itself. Aiming to address the problem of limited resources, Rawassizadeh et al. proposed a resource-efficient data mining framework for small wearable devices [33]. Their framework integrates continuous context sensing and prediction capabilities, both very useful for abnormality detection in AAL applications. They evaluated their framework by implementing it on smartwatch and performing different experiments, involving battery efficiency, memory caching, file storage, semantic abstraction and prediction tools.

### 2.2. Ambient Solutions

Ambient solutions, unlike wearable ones, are less intrusive at least regarding the human body, since they require the installation of sensing elements only inside the home environment. Such solutions, disappearing in the environment, are generally well-accepted by end-users. Conversely, their detection performance depends on the number and careful positioning of sensors, which may require modification or redesign of the home environment. They can include simple switches, or pressure and vibration sensors embedded in carpets and flooring. The latter kind of sensors are particularly useful for detecting abnormal activities, since elderly people are directly in contact with the floor surface during the execution of ADLs. In such a way, a fall event can be detected by considering the occupied area of the floor [34] as well as vibrations or pressure forces [35] involved when the fallen person’s body impacts on the floor surface.

In this regard, Feng et al. proposed a “smart floor” embedded with waterproof fiber sensors sensitive to pressure [34]. Their aim was to investigate the smart floor for detecting falls in a bathroom scenario, where the use of camera-based solutions raise privacy concerns and wearable solutions are not always viable (e.g., when the monitored person is taking a shower). They adopted a threshold-based supervised methodology in which parameters were manually tuned for each involved participant. A different “floor sensing” approach has been investigated by Droghini et al. [35], who employed an acoustic sensor attached to the floor surface to capture the sounds produced by a fall event. The acoustic sensor consisted of a microphone arranged inside a resonant enclosure, acoustically coupled with the floor surface by means of a membrane. The authors demonstrated that their solution can isolate fall-related sounds from those produced by different sources (e.g., voice, music) much better than wall/ceiling-mounted microphones. Regarding the detection methodology, they used an unsupervised approach based on one-class support vector machine (OCSVM) classifier, trained on a large corpus of normal sounds, followed by a template-matching stage whose templates were user-labelled false alarms.

Other widely investigated solutions, also falling into the category of ambient sensing, are based on sensors able to work remotely (i.e., without contacting the sensed target), mounted on wall or ceiling of a room. In the case of acoustic and visual sensing, microphones [36] and cameras [37] are, respectively, used to perform some kind of scene analysis. Li et al. proposed a circular acoustic array including eight omnidirectional microphones [36]. Their computational framework included source localization and height estimation, mel-frequency cepstral coefficients (MFCC) feature extraction and nearest neighbor (NN) classifier for sound event discrimination as fall or not. In their approach, the NN classifier was trained in supervised mode with simulated falls performed by three stunt actors.

Those based on camera are the most highly performing and extensively investigated solutions, although they may raise significant privacy concerns. The camera-based fall detection approach investigated by Rougier et al. consisted of four uncalibrated monocular cameras [37]. To discriminate activities as fall or not, they used a Gaussian mixture model (GMM) classifier trained with normal activities (in an unsupervised fashion), and so they used a decision threshold to discriminate falls as anomalies.

Range sensing is another contactless ambient modality based on the remote measurement of distances. Commonly employed sensors are pyroelectric infrared (PIR) [38,39,40], sonar [41], Lidar/range camera [42,43], radar [44] and Wi-Fi systems [45]. Acceptability and performance are quite good, especially in the case of Range camera and Radar, since depth maps and radar scans are not able to capture privacy-sensitive information.

Iarlori et al. presented a RGBD-based computer vision system for monitoring older adults affected with mild cognitive impairment (MCI), aiming to diagnose the stage of illness by observing ADLs [43]. To this end, they trained a recurrent neural network with parametric bias (RNNPB), in a supervised manner, to recognize a set of sub-action such as “grooming hair,” “washing teeth” and “washing hands.” However, the capturing and processing of RGB data, along with depth ones, do not make the monitoring system fully privacy preserving. Leone et al. suggested an elderly monitoring system, specifically focusing on fall detection via depth camera [42]. They investigated different mountings of depth camera in real-home scenarios, considering also situations in which the floor plane was not entirely visible (e.g., covered with carpets or little objects). The processing of only depth data allowed to demonstrate a fully privacy-preserving monitoring solution. Their detection methodology, however, was based on manually-set thresholds for centroid height and post-fall/recovery phase time duration.

The plethora of ambient sensors seen before would certainly allow extensively instrumenting a home environment to monitor the many events worth detecting in AAL contexts. However, the deployment of so many ambient sensors in each room would be prohibitively expensive. A similar problem has been addressed by Laput et al. who suggested what they called “synthetic sensors” for general-purpose sensing in smart environments [46]. Their processing architecture included a feature extraction layer that converted raw sensor data into an abstract representation. Then, a machine learning layer provided as output a “synthetic sensor,” by abstracting low-level data (e.g., vibration, light color, etc., emitted by a coffee machine) into user-centered representations (e.g., coffee ready sensor).

Among the various ambient sensing technologies, UWB radar might be a good candidate for introducing general-purpose sensing in AAL contexts. In fact, other than being a fully privacy-preserving and unobtrusive technology, it has been already demonstrated in relevant tasks, including detection of vital signs [47], daily activities/behaviors [48], falls [44], tremors [49], walking speed [50], gait events [51], sleep quality [52], and room occupancy [53].

### 2.3. Radar-Based Fall Detection

Su et al. investigated the detection of falls by means of a low-cost (i.e., price comparable to a webcam) CW Doppler radar, having carrier frequency of 5.8 GHz and pulse repetition rate (PRR) of 10 MHz [54]. They exploited time–frequency features extracted via Wavelet Transform (WT), and used supervised detection by comparing NN and support vector machine (SVM) classifiers, trained with a dataset of 21 simulated falls. The better performance was achieved with NN classifier, resulting in 97% sensitivity, 92.2% specificity, and 93% accuracy.

The Doppler signature generated by a moving person was exploited by Wu et al. in their radar-based fall detection system [55]. Such signatures were obtained by considering 4 s windows of the Doppler spectrogram centered around catastrophic events, candidate to be falls. Thus, signatures were treated as images (time–frequency axes), and after some morphological enhancement a feature vector was manually defined. To detect falls, the authors compared two supervised classifiers, SVM and relevance vector machine (RVM), both trained with simulated falls. The experimental setting took place in their laboratory, equipped with an Agilent network analyzer with time sampling rate of 1 KHz and carrier frequency of 8 GHz. RVM and SMV achieved the same accuracy, but RVM required only five relevance vectors against 20 support vectors obtained by SVM. The detection performance was generally good, although fast activities (sit-and-stand, bend-and-stand-up) tended to generate false positives.

The Doppler signature was exploited also by Jokanovic et al. in their interesting work on fall detection using radar sensing and deep learning [56]. The Doppler signature, obtained via short-time Fourier transform (STFT), was treated as input image to the next stage of feature extraction, as done by previous authors. However, they used a deep learning approach (i.e., stacked auto-encoders) to automatically extract features, and a supervised soft-max classifier trained with simulated falls. During experiments, they used a monostatic CW radar realized with an Agilent network analyzer, employing a carrier frequency of 6 GHz and (externally triggered) sampling rate of 1 kHz. The deep learning-based approach showed a success rate of 87%, much better than the other two compared approaches based on manually defined features and principal component analysis (PCA), which achieved success rates of 78% and 83%, respectively.

Erol et al. proposed a time-integrated range-Doppler approach, with the aim to improve the range resolution of CW Doppler radar, given the importance of range information in fall detection [57]. In their experimental setting, they used two radar systems placed on an L-shape geometry, having carrier frequency of 24 GHz, PRR of 1 kHz, and a bandwidth of 2 GHz providing a range resolution of 7.5 cm. The Doppler spectrograms, augmented with range information, obtained from the two radar units were fused comparing three data fusion methods: data-level, feature-level, decision-level. As detection methodology, the SVM classifier with a radial basis kernel function was utilized, trained with simulated falls in a supervised way. The best detection accuracy was 95.95%, achieved with the feature-level data fusion.

### 2.4. Radar-Based Vital-Sign Measurement

Radar-based vital sign sensing caught the interest of researchers from the 70s, when the first experiments were carried out aiming to detect remotely RR [58,59,60] and HR [61] parameters. The measuring principle of vital signs with radar exploits tiny chest movements caused by the respiratory and circulatory motions (contraction and expansion) which induce changes in electromagnetic (EM) wave returning back to the radar system once reflected by the subject’s chest.

Such changes contain information about RR and HR of the subject, and they essentially may occur in terms of frequency, phase, and arrival time of reflected EM wave [62]. The frequency-changing effect is used in the Doppler radar which is one of the earlier radar-based approach for vital sign detection [57], and also successfully adopted for long-range (up to 69 m, in line-of-sight) detection of RR and HR [27,63,64,65]. The phase-changing effect is normally exploited in the interferometric radar, recently demonstrated also for vital sign detection, achieving highly accurate measurements although at the price of a greater complexity and expense [66].

Regarding the third changing effect, i.e., arrival time, it governs the working principle of impulse radar systems which, thanks to generated train of ultrashort EM pulses, can operate over a larger bandwidth and wider range of frequencies than CW systems. UWB-IR radars, together with Doppler radars [67], are the most investigated for physiological function monitoring [68]. Since Doppler radars are typically CW narrowband systems, they can accurately measure the velocity of targets (i.e., high Doppler resolution) but not their position (i.e., low range resolution), making it difficult the cancelation of motion artefacts caused by the subject or by other nearby people as well as the detection of vital signs from more than one person. Conversely, the UWB-IR radar offers a much higher range resolution, carrying useful information for estimating vital signs even when multiple people are present.

Li and Lin addressed the problem of vital sign detection with Doppler radar in presence of random body movements [69], which generally prevent the accurate detection. For this purpose, they investigated two different signal demodulation methods, namely complex signal demodulation and arctangent demodulation. In their experiments and simulations, they demonstrated that the first method is robust when the dc offset is available and can be implemented easier. The second one can be user also without dc offset, and allows to eliminate both harmonic and intermodulation interferences at high frequencies. However, these methods require the use of two identical radars for detecting the human body from both front and back sides.

Recently, Hu and Jin investigated the UWB-IR radar in detecting HR and RR, by using ensemble empirical mode decomposition (EEMD) and continuous-wavelet transform (CWT) [70]. After a clutter removal stage, EEMD was used to decompose the signal into its intrinsic mode functions (IMFs), thus obtaining a noise reduction effect. After that, the CWT was used to separate the vital signs, HR and RR.

For the sake of completeness, it is worth noticing that remote detection of vital signs can be achieved by using not only radar, but also optical sensing through camera-based photoplethysmography (cbPPG). The working principle of cbPPG is to detect small changes in the skin color due to cyclic variations of blood volume in arteries and capillaries under skin, and thus to estimate the PPG signal which is proportional to such skin color changes [71]. The cbPPG technique, unlike the radar-based one, allows to estimate also the blood oxygen level (SpO2). However, radar sensing is more accurate in estimation of RR and HR, particularly in presence of multiple heartbeats and cluttered scenarios with obstacles [72]. Furthermore, it is a fully privacy-preserving sensing technology, since captured information is outside the human sensory capabilities (unlike cbPPG and cameras in general that capture images), and thus not directly usable for obtaining privacy-sensitive information.

## 3. Materials and Methods

The purpose of this study was to develop and validate a Radar Smart Sensor (RSS) able to detect both cardiorespiratory and body movements without causing any discomfort to older adults. In the remainder of this section, the system architecture is gradually detailed, starting with a general overview and then describing each system parts, with major focus on micro-Doppler processing, micro-movement signature definition, and vital signs estimation via Empirical Mode Decomposition (EMD). Finally, the experimental setup and validation procedure are presented.

### 3.1. System Overview

**Figure 1:**
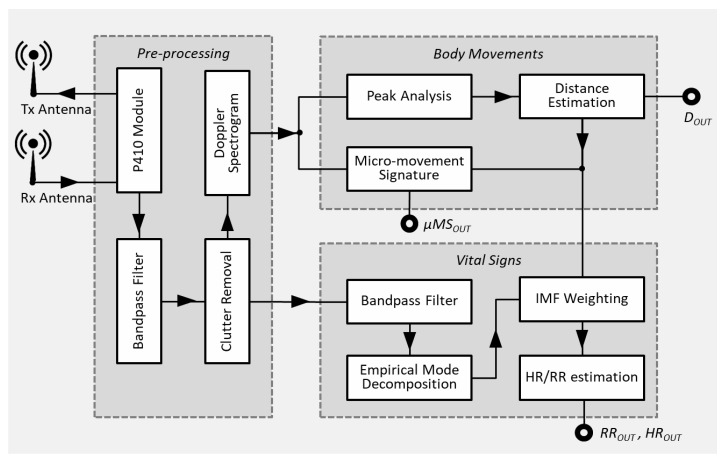
RSS system overview.

The detection system, of which a schematic representation is given in Figure 1, is composed of the three main stages: (1) pre-processing; (2) body movements; and (3) vital signs. The pre-processing receives signals from the radar unit (i.e., the P410 module) and provides signal processing functions useful for the other two stages of the systems. The “body movements” stage is devoted to the computing of micro-motion signatures (*μMS*) and distances between body and antenna (*D*), starting from the Doppler-spectrogram provided by the “pre-processing stage”. The third stage, “vital signs”, received the clutter-free signal as input estimates the HR and RR, using also the distance information computed by the “body movements stage”. The RSS shown in Figure 1 was assembled as two independent parts. The P410 was provided with its own board. Instead, all other blocks were implemented on the embedded pc (EPC) described in Section 3.5. All aforementioned main stages are further detailed in the following sections.

### 3.2. Pre-Processing

#### 3.2.1. Radar Module

Radar systems can be categorized based on their radio-wave bandwidth into: narrowband (NB) and UWB. The UWB is a radio technology using either pulse (IR) or CW of very short duration, and operating on frequency range wider than 500 MHz or 25% of the center frequency. More specifically, the UWB-IR, operating over a larger bandwidth and wider range of frequencies [20], provides additional features over UWB-CW, particularly useful in AAL (Ambient Assisted Living) contexts. The sub-millimeter range resolution and high penetration power enable the detection of very small target event through obstacles (e.g., through-wall sensing of vital signs). The shorter pulse duration, lower than the total travel time of the wave even in case of multiple reflections, is helpful to deal with multipath effects particularly insidious in indoor environments. The very low power spectral density prevents interferences with other radio systems operating in the same frequency range, and guarantees a low probability of interception; enabling secure high-data-rate communication in short range (e.g., up to 500 Mbps at 3 m).

**Figure 2:**
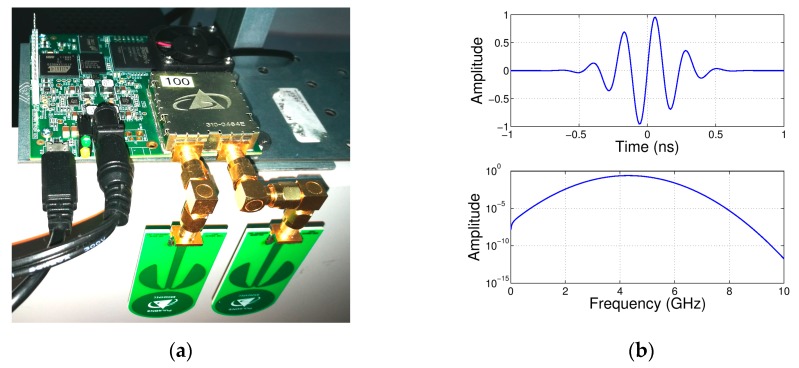
(**a**) P410 radar module; and (**b**) P410 Pulse waveform (top-side) and related frequency spectrum (bottom-side).

The Time Domain PulsON P410 [73], reported in Figure 2a, is a state-of-the-art UWB-IR radar module, enabling precise measurements in high multipath and high clutter environments. The P410 is characterized by low cost, small size (7.6 × 8.0 × 1.6 cm board dimensions), as well as low power operation (from −33 dBm to −13 dBm) conforming to FCC requirements; all made possible by a dedicated UWB chipset, which includes various software-configurable parameters useful for application customization. The pulse waveform is a bandpass signal with frequency spectrum 3.1–5.3 GHz centred at 4.3 GHz, as exemplified in Figure 2b, generated at a pulse repetition rate of 10 MHz, and received at sampling rate of 61 ps.

**Figure 3:**
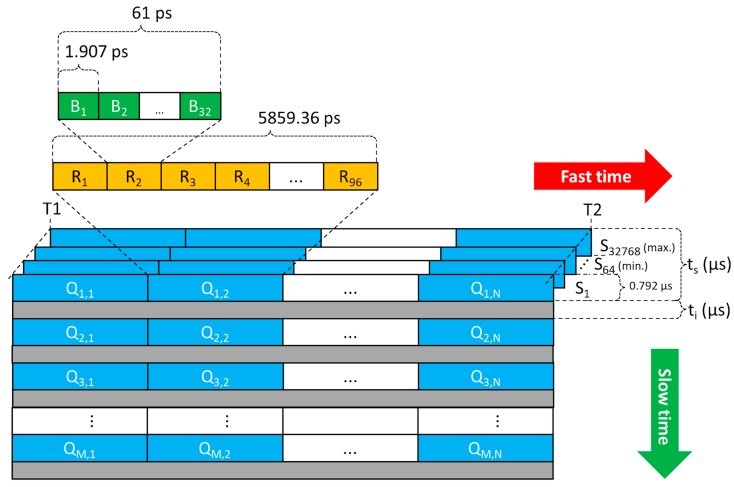
Monostatic Radar Module (MRM) receiver architecture of the radar module.

The Monostatic Radar Module (MRM) receiver architecture of P410 is represented in Figure 3. As seen from this figure, the radar scan data are converted into 32 bins (i.e., the green-colored cells in Figure 3), having time duration of 1.907 ps (i.e., the fast-time sampling is 31 × 1.907 ps ≈ 61 ps), and then they are stacked into a stack segment of 96 bins covering a total time of 5859.36 ps (i.e., the orange-colored cells in Figure 3).

The distance range covered by the radar is associated with a time-axis, known as fast-time (i.e., the red colored arrow in Figure 3), expressed in the order of nanoseconds. Conversely, the time-axis along to the sampling interval is called slow-time (i.e., the green colored arrow in Figure 3) and expressed in microseconds. As well known, the relation between distance R and total travel time T is the following: R=c ∗ T/2, where c is the speed of the light in vacuum. However, T cannot be chosen at will, but must be quantized according to timing constraints imposed by the MRM architecture. More accurately, given a desired distance range interval $\left[\tilde{R1},\;\tilde{R2}\right]\;$(with $\tilde{R1}<\tilde{R2}$, in meters), the actual range interval representable within the RMR will be [R1, R2] that can be estimated as follows. Firstly, given $\tilde{R1}$ and $\tilde{R2}$ the total number of scan bins *N* (i.e., blue colored cells in Figure 3) can be obtained by considering that each stack bin has a time duration of 5.8594 ns and is further subdivided into 96 bins, thus:

$$N\;=\;96\lceil 2\frac{\tilde{R2}\;-\;\tilde{R1}}{5.8594\;c}\rceil ,$$

where the angled brackets ⌈ · ⌉ indicate the ceiling operator, and c = 0.29979 m/ns. Secondly, the fast-time instant T1 corresponding to R1 can be empirically estimated as follows:

$$T1\;=\frac{\left\lfloor 1.9073\lfloor \frac{1000\;}{1.9073}\;\left(\frac{2\;\tilde{R1}}{c}+10\right)\;\rfloor \right\rfloor }{1000},$$

where T1 is expressed in ns, the angled brackets ⌊·⌋ indicate the flooring operator, 1.9073 ps is the time duration of a radar scan bin, and 10 ns is the minimum fast-time instant accepted by the RMR. Similarly, the instant T2 (in ns), corresponding to R2, can be empirically estimated as follows:

$$T2=\frac{\left\lfloor 1.9073\lceil 32\;N+\frac{1000\;}{1.9073}T1\;\rceil \right\rfloor }{1000}.$$

Finally, the actual range interval [R1, R2] can be estimated as follows:

$$R1=\frac{c}{2}\left(T1-10\right),\;R2=\frac{c}{2}\left(T2-10\right).$$

Since the receiver architecture is based on several parallel samplers (i.e., rake receiver), it allows the integration of multiple scans $S_{k}$ in order to improve the SNR (Signal-to-Noise Ratio) of radar returns. The minimum number of integrated scans is 64 (i.e., 2^6^) corresponding to a SNR increase of 18 dB which further increases of 3 dB at each doubling of integrated scans, up to a maximum of 32,768 (i.e., 2^15^) scans, i.e., 45 dB. The time duration $t_{s}$ of a full scan depends on two factors: (1) the number of integrations given by $2^{\mathrm{PII}}$, where PII (Pulse Integration Index) spans from 6 to 15; and (2) the distance range, i.e., the size of the scan window, given by T2−T1. Hence, $t_{s}$ (expressed in μs) can be estimated as follows:

$$t_{s}=0.792\;\times \;2^{\mathrm{PII}}\;\times \;\mathrm{Round}\left(\frac{T2-T1}{5.8594}\right),$$

where T1 and T2 are in ns, 5.8594 ns is the time duration of each stack segment, and 0.792 μs is the time duration of a scan in slow-time (see Figure 3, green arrow direction).

In addition, between one scan and another, there is a further time interval $t_{i}$, so that the (slow-time) sampling frequency is given by $F_{s}=t_{s}+t_{i}$. In the present study, the MRM parameters were selected in order to cover a distance range varying from 0.5 m to 5.77 m, at sampling frequency of $F_{s}=50$ Hz and with 36 dB of increase in the SNR (i.e., PII = 12). For this purpose, $\tilde{R1}$ was kept fixed at 0.5 m and $\tilde{R2}$ was increased from 1.5 to 5.5 at steps of 1 m. The corresponding RMR parameters, estimated as said above, are reported in Table 1.

#### 3.2.2. Bandpass Filtering

Interference and noise due to various types of sources may cause undesirable signal degradation. In presence of wideband sources, the related noise has the form of short random pulses which can be significantly attenuated by integrating (and averaging) multiple received signals, thanks to the, previously described, rake receiver architecture. Instead, in the case of narrowband sources, which mainly are nearby systems generating electromagnetic interference with sinusoidal waveform and random amplitudes, usually a bandpass filtering is used to attenuate this type of noise. To this end, in the present study, the received radar signal was filtered by a 16th-order Butterworth with bandpass in the radar operating frequency range, i.e., from 3.1 to 5.3 GHz.

The filter order was obtained by considering a max. passband ripple of 3 dB and attenuation in stopband of 30 dB. Then, the stopband width was gradually decreased starting from 1.5 GHz (i.e., 3rd-order Butterworth), while measuring the time delay (execution time) due to the filtering processing. The processing workload was evaluated by filtering a radar scan at the maximum range (i.e., *N* = 576 bins) on the reference computing platform reported in Section 3.5. A good compromise was found with 16 ns delay and 230 MHz stopband width, corresponding to a 16th-order Butterworth [74].

#### 3.2.3. Clutter Removal

Beside noise and interference, the clutter is another problem which may reduce the SNR of radar returns. The clutter returns are unwanted signal components induced by reflection from static structures included in the environment (i.e., walls, furniture), and whose energy can be several orders magnitude larger than the useful signals reflected from the person’s body (e.g., torso, limbs, chest cavity, etc.).

In the past years, many clutter removal techniques have been investigated, which can be roughly classified as background subtraction [75], filtering [76], wall-parameter modeling [77], statistical approaches [78], and nonlinear approaches [79]. Among these techniques, background subtraction, filtering and wall-parameter modeling are not particularly versatile, since they require underlying assumptions, such as, on either background scene (free of moving objects) or spectrum bandwidths (wall and target reflections) [80]. Nonlinear approaches are the most general, but they are also quite computationally expensive owing to the iterative nature [79]. Conversely, statistical approaches are the most interesting ones, since they have low computational complexity and often exhibit feature extraction capabilities.

Verma et al. compared some of the most promising statistical approaches, namely principal component analysis (PCA), independent component analysis (ICA), factor analysis (FA) and singular value decomposition (SVD) [78]. The ICA approach gave the better result, in particular for through-wall imaging of low-dielectric targets. Instead, PCA, SVD and FA performed in a quite similar way. In this study, the SVD-based clutter-removal was chosen for its low computational cost and simplicity over the other approaches. Following this approach [81], the signal matrix was SVD decomposed obtaining a diagonal matrix whose first “few” descending-ordered singular values conveyed the largest amount of clutter energy. By setting these singular values to zero and reconstructing the signal matrix, the clutter energy was removed and the SNR improved.

#### 3.2.4. Micro-Doppler Spectrogram Processing

In radar sensing, the velocity of the moving target can be obtained by exploiting the Doppler effect, based on which the frequency of the received signal is shifted from the frequency of the transmitted signal [82]. The Doppler frequency shift is proportional to the radial (i.e., in the direction of the line of sight) velocity of the target: it is positive if the target approaches the radar, and negative if the target moves away. Thus, when the target is not a rigid body but has several parts characterized by an oscillatory motion (e.g., a walking human), such oscillation produces an additional Doppler frequency modulation called micro-Doppler effect [82]. Such micro-Doppler modulation can be regarded as a distinctive signature able to account for unique properties of a target. More specifically in this study, the micro-Doppler signature is exploited to detect, localize and track a monitored person, as well as to discriminate normal activities from abnormal ones, such as falls.

**Figure 4:**
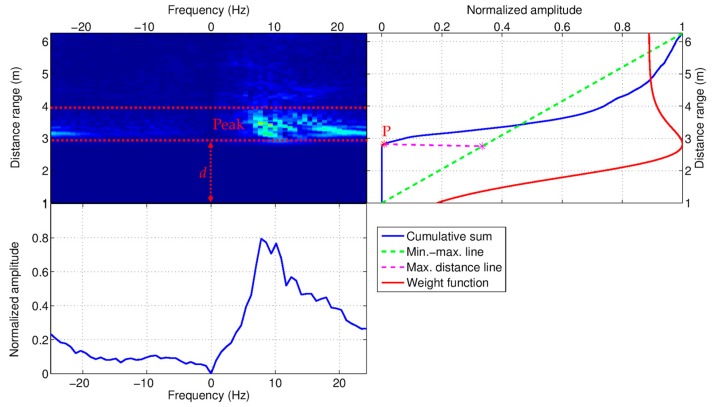
Doppler spectrogram (**top-left** image) from which the body position (i.e., distance from the radar antenna) is estimated (**top-right** image), and the micro-motion signature extracted (**bottom-left** image).

The Doppler spectrogram was used first for estimating the distance of the person’s body from the radar, and then for extracting the micro-motion signature useful for both person localization and activity recognition. The body position was estimated by projecting the spectrogram on the distance range. After that, the micro-motion signature was obtained by projecting the Doppler spectrum on frequencies, but restricted to the only region of the distance range including the estimated body position. Both procedures are exemplified in Figure 4. The Doppler spectrogram was computed by applying the discrete-time Fourier transform (DTFT) to the analytic version of the clutter-free signal, i.e., the output signal provided by the clutter removal module. As well known the analytic signal is a complex signal obtained by setting the imaginary part to be equal to the Hilbert transform of the original real signal [83]. The DTFT length was fixed to N=16, for computational efficiency reasons, to which corresponded a time duration of $T_{\mathrm{DTFT}}=\frac{N}{\mathrm{Fr}}=$ 320 ms by considering a short-time sampling frequency of Fr = 50 Hz. As an example, the Doppler spectrum related to a walking action is depicted in Figure 4 (top-left image).

### 3.3. Body Movements

As discussed in Section 3.2.4, the Doppler spectrogram provides information about human movements in the form of micro-Doppler signature. In this work, the micro-Doppler signature was extracted by means of a two-step procedure. The first step was to estimate the distance d between the (closest) person and the radar sensor. The second step was to extract a micro-motion signature by considering the only spectrogram region located beyond the estimated distance d. Referring to Figure 4, to estimate the person-radar distance d, the spectrogram was projected on the distance axis (i.e., *y*-axis) by taking the cumulative sum over distances of the spectrogram image. An example of this is shown in Figure 4 (right hand side) where the cumulative sum is plotted as solid blue line. The property of the cumulative sum it to rise in correspondence to the spectrogram peak region, hence it can be exploited to identify the peak region. To this end, the point P of maximum curvature change of the cumulative sum was estimated. Referring to Figure 4, the point P is the farthest point from the line joining the minimum and maximum points (green dotted line). Then, the distance d was estimated as projection of P on the distance axis (d≅3 m in the example of Figure 4).

Since, in general, the spectral content is not uniformly spread over the spectrogram, but, on the contrary, is confined in the range region interested by the body’s movement (e.g., the range region between 3 m and 4 m in Figure 4), to improve the SNR, a special sigmoidal-shaped function was considered (solid red line in the top-right part of Figure 4) having the following analytical expression: $h\left(x\right)=4\frac{1\;+\;\tanh x}{{\left(2\;+\;\tanh x\right)}^{2}}$. The property of this function is that it does not decay uniformly after the curvature change but instead it maintains a high gain level for a while, after that it rapidly declines to a constant value. Then, the spectral region related to body’s movements was filtered by multiplying the spectrogram by the response function H(f,x)=h(x−d) with f varying in frequency domain and x in distance range. Finally, the micro-motion signature μ-MS was extracted from the filtered spectrogram as average summation over frequencies (solid blue line in the bottom-left part of Figure 4). The average was calculated by taking $N_{S}$ (50%-overlapped) DTFTs, thus covering a time window of $T_{S}=\frac{N_{S\;}+\;1}{2}T_{DTFT}$ per signature. The optimal $N_{S}$ was determined using ROC (Receiver-Operating Characteristic) analysis.

It is important to note that the combined peak analysis of both micro-motion signature and distance d (over fast-time and slow-time, respectively) allowed to discriminate the regions of the spectrogram in which body movements were more intense from those where they were less intense, and thus provided an effective strategy for movement compensation during the estimation of vital signs.

### 3.4. Vital Signs

The SNR of radar returns reflected from the monitored subject’s chest, useful for vital signs estimation, can be affected by unwanted signals or noise (e.g., generated by periodic sources such as fans, motors, curtains/doors motion, etc.). To attenuate this inconvenience, in this study, a second bandpass filter was implemented, namely a 6th order IIR Butterworth in slow-time with a passband from 0.125 Hz (corresponding to a minimum of 7.5 breaths/min) to 3 Hz (corresponding to a maximum HR of 180 beats/min). The filter order was defined by following a strategy similar to that already described in Section 3.2.2, aiming to find a good trade-off between computational complexity and filter selectivity. In this case, the achieved stopband width was of 0.8 Hz and processing time of 44 ns for filtering 1 min radar scans (i.e., 3000 scans), within a distance range of about 50 cm (i.e., 54 bins), with respect to the reference computing platform reported in Section 3.5.

As it is well-known, the human cardiorespiratory system is characterized by thorax displacements due to respiration activity larger than those due to heartbeat (i.e., at least 12 mm against only 0.6 mm) [84]. Hence, although the heart and respiration signals are normally separated in frequency domain, it is difficult to isolate them using traditional filtering techniques. To this end, in fact, the respiration signal should be attenuated over 50 dB more than the heart one [85].

In recent years, time–frequency analysis has been increasingly investigated to separating heart and respiration information carried by the radar signal. Commonly used time–frequency analysis methods include STFT [86], CWT [87], and Chirplet Transform (CT) [88]. However, such methods are formulated in terms of integral transforms and analytic representations, giving rise to some practical limitations affecting their effective usage in real-world applications [89]. To overcome such limitations, EMD and Hilbert-Huang transform [90] have been recently heralded as promising time–frequency analysis methods for separating time-varying and nonlinear signal components, in various application domains including radar sensing of vital signs [91,92,93,94].

In this study, the EMD method was used to decompose the filtered radar signal into its intrinsic signal components, including heart and respiration signals. The purpose of EMD is signal decomposition into intrinsic components, likewise the Fourier method. However, such intrinsic components, more properly called intrinsic mode functions (IMFs), are not required to be sinusoidal, instead they have well-defined instantaneous frequencies.

As it is well documented in literature [89,90], the EMD method is an iterative algorithm consisting of three main steps, known as sifting process, repeated iteratively until a specific stop-criterion is satisfied (introduced below). Given the signal s(t) being decomposed, the three main steps are as follows:

(1) The upper $e_{U}\left(t\right)$ and lower $e_{L}\left(t\right)$ envelopes of s(t) are estimated, by interpolating with cubic splines the local maxima (upper envelope) and local minima (lower envelope) of s(t).
(2) The mean of the two envelopes is calculated: $m\left(t\right)=\frac{e_{U}\left(t\right)+e_{L}\left(t\right)}{2}$
(3) The local high-frequency signal ℓ(t) is obtained as ℓ(t)=s(t)−m(t).

**Figure 5:**
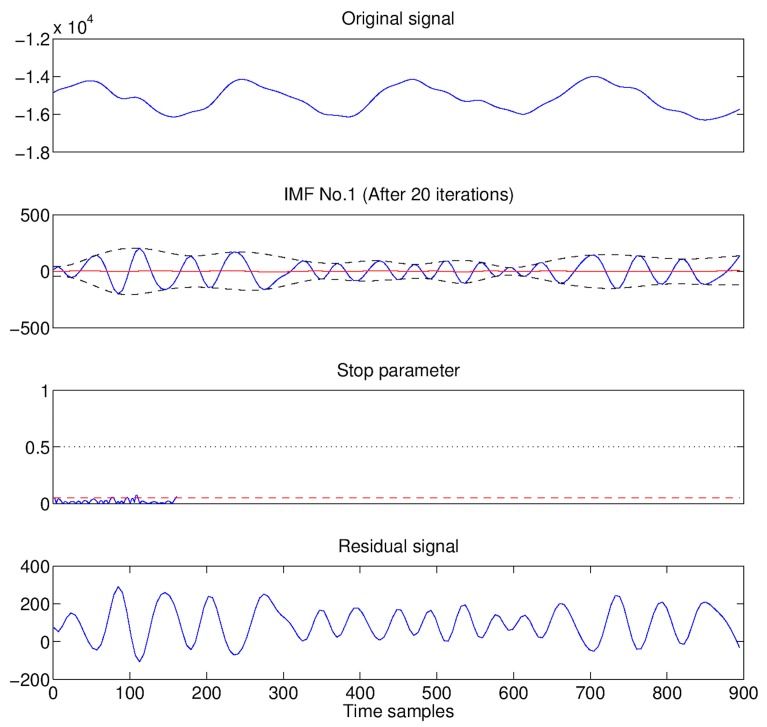
Extraction process of the first Intrinsic Mode Function (IMF) (heart signal) from a radar return via Empirical Mode Decomposition (EMD) procedure.

At each iteration, ℓ(t) is checked against a stop-criterion that consists of two conditions [95]: (a) the number of local extrema of ℓ(t) differs from the number of zero-crossings of ℓ(t) by at most one; and (b) the mean m(t) is close to zero, i.e., it drops below a given threshold. If the stop-criterion is satisfied, the current ℓ(t) is an IMF, and the sifting process restart with the new signal $s^{\prime }\left(t\right)=m\left(t\right)$. As an example, in Figure 5, the extraction via EMD of the heart signal (as first IMF after 20 iterations) from a radar return is reported.

As seen in Section 3.2.4, the body movements interest only a limited portion of the distance range. Let D={d | D1≤d≤D2} be the current, or the latest region, in which movements (closest to the sensor) have been detected. For example, referring to Figure 4, the interested range is delimited by D1 = 3 m and D2 = 4 m. Given an observation time window of size T, a certain number of radar scans $s_{k}$, confined in D, were decomposed via EMD in $n_{k}$ modes, namely $M_{k}=\left\{f_{k,j}\;|\;\forall j=1,\ldots ,n_{k}\right\}$.

To be able to estimate HR and RR from extracted modes, a weight $w_{k,j}=\|\mathrm{pdf}\left(s_{k}\right)-\mathrm{pdf}{\left(f_{k,j}\right)\|}_{2}$ was assigned to each $f_{k,j}$, where pdf(·) is the probability density function (PDF), std(·) the standard deviation, and ${\left\|\cdot \right\|}_{2}$ the L2-norm. The choice of L2-norm is motivated by the study of Komaty et al. [96], who investigated several similarity measures in identifying relevant modes of a signal. Their results showed that the L2-norm was the most efficient. The PDFs were approximated by histograms having bin-size equal to $\max\left\{\mathrm{std}\left(s_{k}\right)\;|\;\forall k\in K\right\}/4$. The weight sequence $\left\{w_{1,1},w_{1,2},\ldots ,w_{1,n_{1}},w_{2,1},w_{2,2},\ldots ,w_{2,n_{2}},\ldots \right\}$ was used to identify the IMFs that best described the signal (i.e., the cardiorespiratory signal).

**Figure 6:**
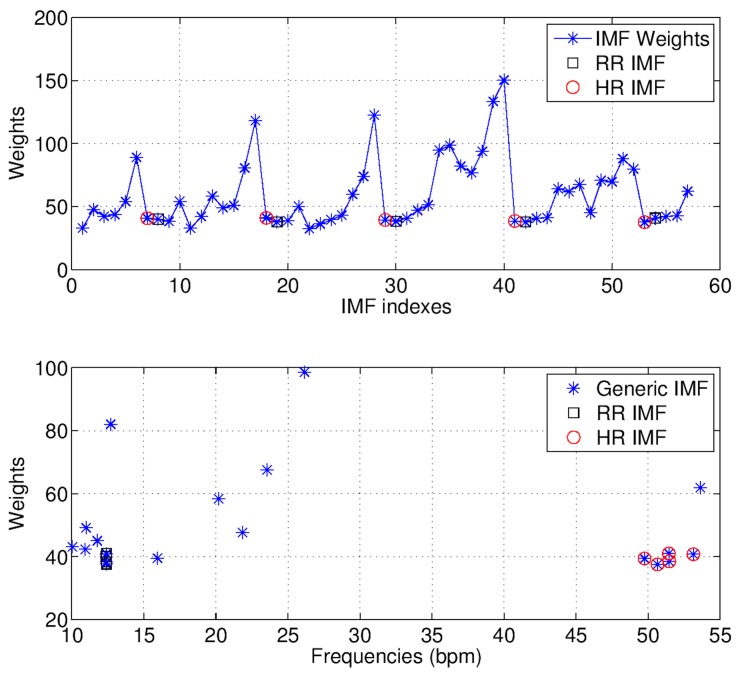
Selection strategy of the IMFs which best describe the cardiorespiratory signal. The selected IMFs are related with the minimum weights immediately following the local maxima (the **upper** plot in this figure). Finally, the heart rate (HR) and respiration rate (RR) are estimated as average frequencies of the selected IMFs (the **lower** plot in this figure).

The IMF selection strategy can be intuitively explained by observing the “up and down” behavior of the weight sequence, as illustrated in the example shown in Figure 6. For each scan $s_{k}$, the related weight subsequence $\left\{w_{k,1},w_{k,2},\ldots ,w_{k,n_{k}}\right\}$ increases until the last noisy mode (local maximum), then it decreases until its minimum value corresponding to the IMFs that best describe the signal.

### 3.5. Experimental Setup

**Figure 7:**
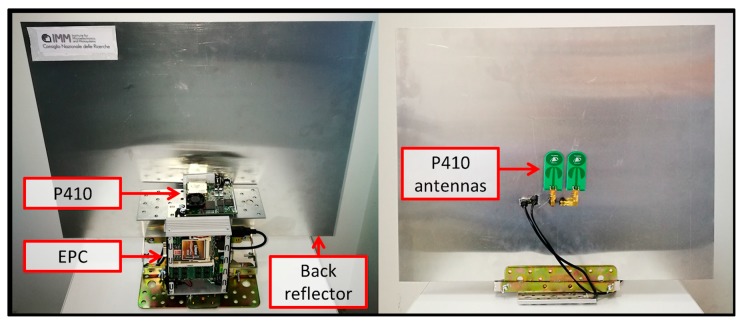
Final assembly of the Radar Smart Sensor (RSS), suitable for tripod mounting.

All previously discussed processing modules were developed in C language and ran on an EPC, with 1.6 GHz Intel^®^ Atom™ Processor Z530 and 2 GB RAM, namely the eBOX530-820-FL manufactured by Axiomtek [97], having compact dimensions of about 132 × 95.4 × 47.5 mm and low power consumption of 25 W. Both EPC and radar module were assembled together into a unique compact structure including also a back reflector to the radar antennas which reduced the azimuth pattern to around 100° (i.e., detection restricted to a zone at the anterior of the antenna). A picture of the resulting RSS is shown in Figure 7.

The experiments were conducted in the laboratory setting by involving 30 healthy subjects divided into two age groups of average 25 and 47 years old, respectively. Each participant simulated various types of ADLs, such as cooking, preparing meals, washing dishes, eating at the kitchen table, sitting on the couch watching TV, resting in bed, doing physical activities. The aforesaid ADLs were grouped in 15 sequences of 900 s (15 min) in duration per participant. Additionally, after each sequence, the participants performed various falls in four different directions, i.e., forward, backward, lateral left/right, as suggested by Nuory et al. [98]. To this end, the two participant groups were separately instructed by geriatricians on how falls should be realistically simulated. Hints from studies on real-life fall events were also taken into account [99,100]. This simulation protocol, which included also the use of protective devices such as padded mat and knee protection, was preventively approved by the local ethics committee.

**Figure 8:**
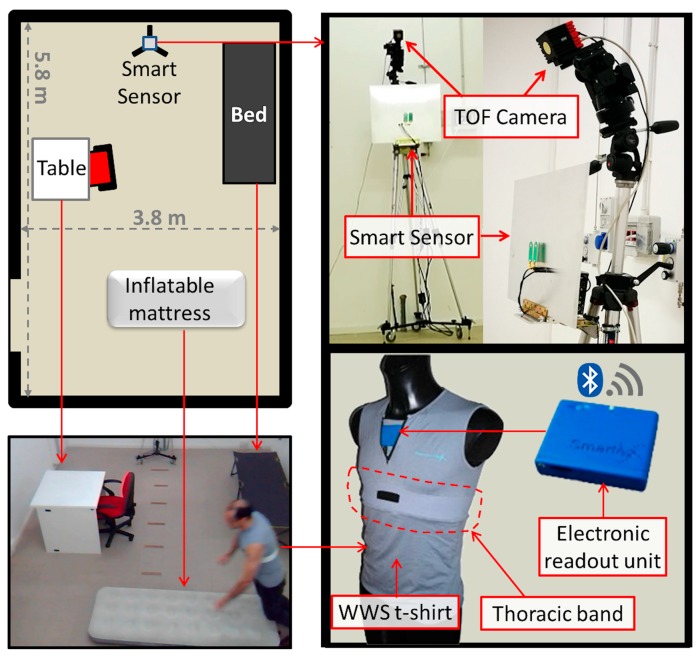
Experimental setup used for validating the RSS. A laboratory room (**left-hand side**) was equipped with movable furniture parts needed to simulate common ADLs and fall events. Ground-truth data were obtained using a Time-Of-Flight (TOF) camera and a WWS t-shirt (**right-hand side**).

As reported in Figure 8, data collection was performed in a laboratory room of about 5.8 m × 3.8 m equipped with the following furniture parts: table, chair, bed and inflatable mat. All furniture parts were easily movable, allowing to simulate ADLs and falls at different distance from the RSS within a distance range of about 5 m and different orientations. The RSS was mounted on a tripod at the far end of the room, at two different heights above the floor, namely 1.20 m and 2.40 m.

### 3.6. Validation

The detection performance was also evaluated in the presence of multiple, moving people. For this purpose, the acquisition sequence was approximatively divided into two parts: during the first part the participants stayed alone in the room, whereas, during the second part, up to five people entered progressively in the room. To obtain the ground-truth data, two additional equipment were used: (1) a Time-Of-Flight (TOF) camera mounted on the same tripod together with the RSS at the height of 3.00 m above the floor; and (2) a sensorized t-shirt worn by each participant.

The TOF camera, SwissRanger SR4000 (MESA Imaging AG, Zurich, Switzerland) [101], was used to accurately capture information about person’s position and movements inside the room, and to automatically annotate starting and ending time of each simulated action, i.e., change of body posture, as well as the occupancy level of the room (i.e., people counting). The SR4000 is a state-of-the-art TOF-based depth camera, having small dimensions (65 × 65 × 68 mm) and noiseless operation, able to provide QCIF (176 × 144 pixels) depth maps at high frame rate (up to 50 fps) within a wide Field-of-View (FoV) (69° × 56°) and long distance range (up to 10 m). Depth maps provided by the SR4000, after conversion into 3D point clouds, were used to automatically detect and count people present in the laboratory room. For this purpose, a high-performing approach was used, able to detect and track all persons’ location at the same time based on an agglomerative clustering method (i.e., tracking-free approach) [102]. Secondly, starting and ending times of each performed action were automatically identified by decomposing (classification task) the action into a sequence of hierarchical postures [103] (starting from four basic postures, namely, standing, bending, sitting, lying down) based on high-discriminative features extracted from point clouds [104].

Regarding the ground-truth of cardiorespiratory data, during the data collection, each participant was wearing a WWS (Wearable Wellness System) t-shirt manufactured by Smartex [105], as shown in the bottom part of Figure 8, equipped with various sensors which provided precise measurements for HR and RR, thanks to the presence of a thoracic band including two textile ECG electrodes and one respiration sensor. In addition, the WWS t-shirt is equipped with a tri-axial accelerometer which provided information about body’s movements useful to supplement that obtained using the SR4000 TOF camera. The HR and RR data measured by the SSS, during the experiments, were validated by comparing them with those measured by the WWS t-shirt. Such comparison was drawn in terms of accuracy measure, defined as the complementary of the mean relative error (MRE) given by $\mathrm{MRE}=\frac{1}{N}\sum_{n=1}^{N}\frac{\left|M_{n}^{SSS}-M_{n}^{WWS}\right|}{M_{n}^{WWS}}$, where $M_{n}^{SSS}$ and $M_{n}^{WWS}$ were the n-th measurements of M, which might be either HR or RR, provided by the SSS and WWS, respectively.

Regarding the micro-motion signature, the RSS was validated against a typical assisted living application, i.e., fall detection. For this purpose, the micro-motion signatures captured during the experiments were analyzed using two main approaches [22]: supervised and unsupervised. The supervised one is the traditional approach for fall detection, in which a classifier is trained with both positive (i.e., simulated falls) and negative (i.e., ADLs) events. Since it is not realistic to assume that the classifier could be trained with falls simulated by end-users, normally, the classifier is trained and tested with falls simulated by people having very different physical characteristics. In this study, the classifier was trained with falls simulated by individuals belonging to the young group, and tested with falls simulated by the older group. The unsupervised approach aims to overcome the lack of (real) fall data in training process, by considering falls as anomalous events. In such a way, the system can be trained to recognize “normal” events from sensor data captured during the end-user’s ADLs, whereas falls are detected as anomalies, i.e., events diverging from the observed “normal” behavior. In this case, during validation, the same participant can be involved in both training (simulating ADLs) and testing phases (simulating ADLs and falls). In this study, for both supervised and unsupervised approaches, a one-class Support Vector Machine (OCSVM) classifier [106] with Radial Basis Function (RBF) kernel was used and trained either with simulated falls or ADLs, respectively.

The fall detection performance was evaluated in terms of true positive rate (TPR) (or sensitivity) and true negative rate (TNR) (or specificity) measures [98], which definitions are based on the counting of true positives (TP), true negatives (TN), false positives (FP) and false negatives (FN), as follows: $\mathrm{TPR}=\frac{TP}{TP+FN}$, $\mathrm{TNR}=\frac{TN}{TN+FP}$. Furthermore, the ROC analysis was performed to determine the best performance at the varying of all the relevant parameters, such as those related with micro-Doppler spectrogram and OCSVM classifier.

## 4. Results

**Figure 9:**
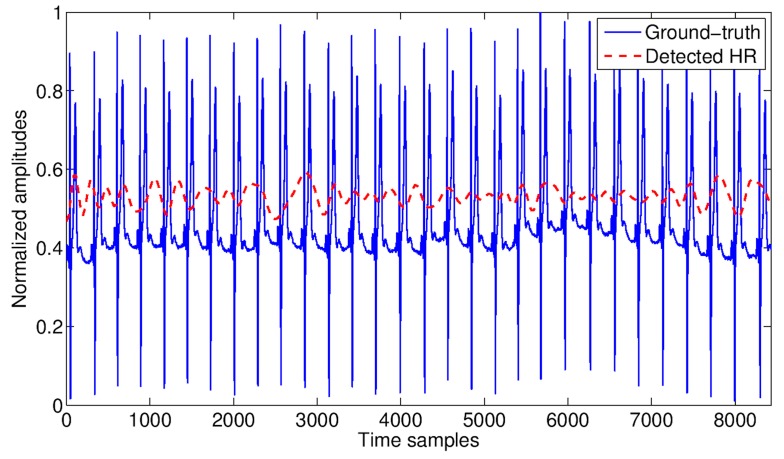
HR measured by the WWS (blue solid line) and by the RSS (red dashed line) via EMD at the distance person-sensor of 2 m. The peaks appearing in the ground-truth signal correspond to the “R” waves.

**Figure 10:**
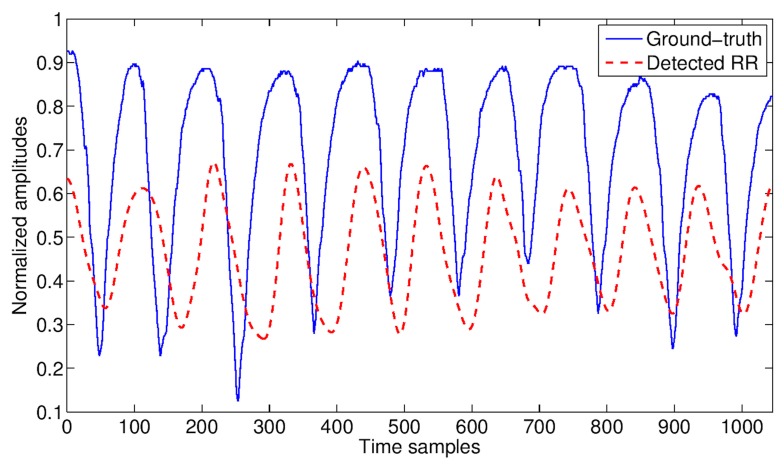
RR measured with WWS (blue solid line) and by the RSS (dashed line) via EMD at the distance person-sensor of 2 m.

The cardiac and respiratory signals detected by the RSS are reported in Figure 9 and Figure 10, respectively, together with the ground-truth, for a qualitative comparison.

**Figure 11:**
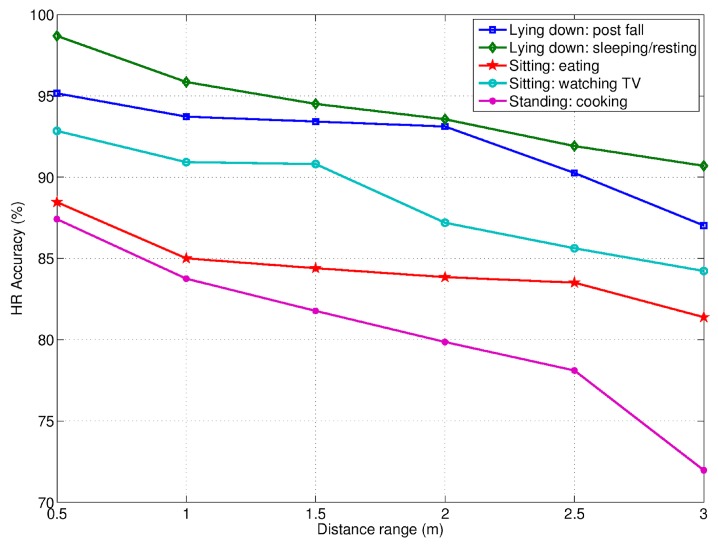
Accuracy of HR detection at varying of distances and ADLs. The only monitored subject was present in the scene.

**Figure 12:**
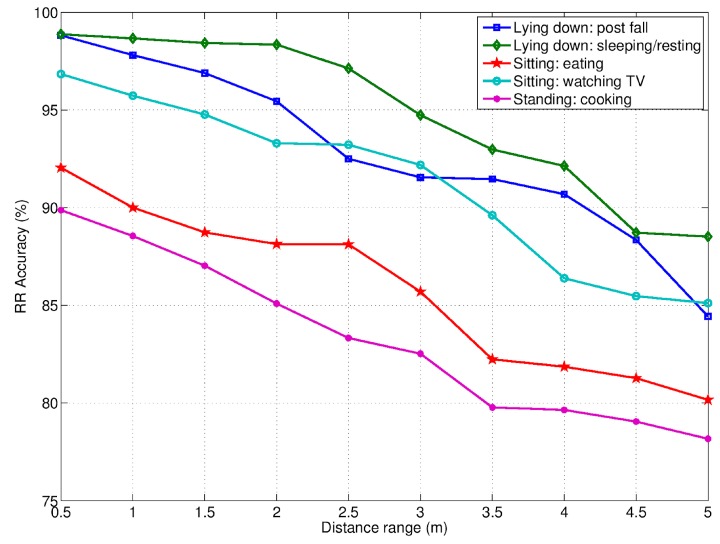
Accuracy of RR detection at varying of distances and ADLs. The only monitored subject was present in the scene.

The quantitative evaluation of HR and RR, in terms of accuracy is shown in Figure 11 and Figure 12, respectively. Here, the accuracy is reported at different distances from the RSS, in correspondence of five different activities, and in presence of the only monitored subject (i.e., only one person in the room). The average HR and RR accuracies for each activity as well as fall event are summarized in Table 2.

**Figure 13:**
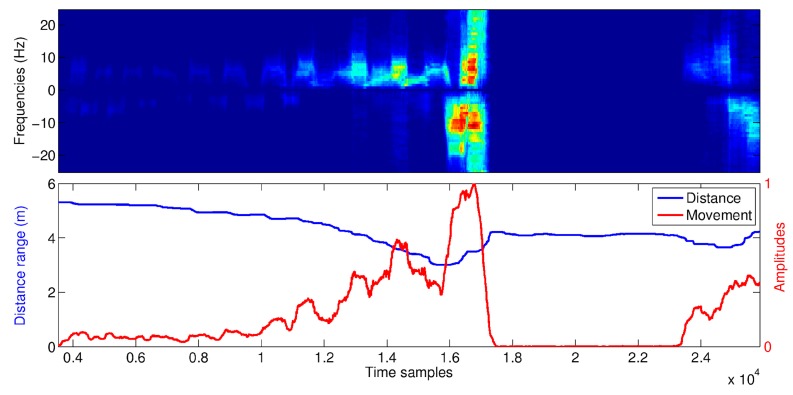
Micro-motion signatures (**top side**), distances and movement amplitudes (**bottom side**) detected by the RSS during a validation sequence including a fall event (starting at the time sample T = 16,000).

In Figure 13, a sequence of micro-motion signatures related to a fall event is reported, together with the distance information and the movement amplitudes. The fall event occurs between time samples 16,000 and 17,000. The activity performed before the fall event is walking at increasing speed.

**Figure 14:**
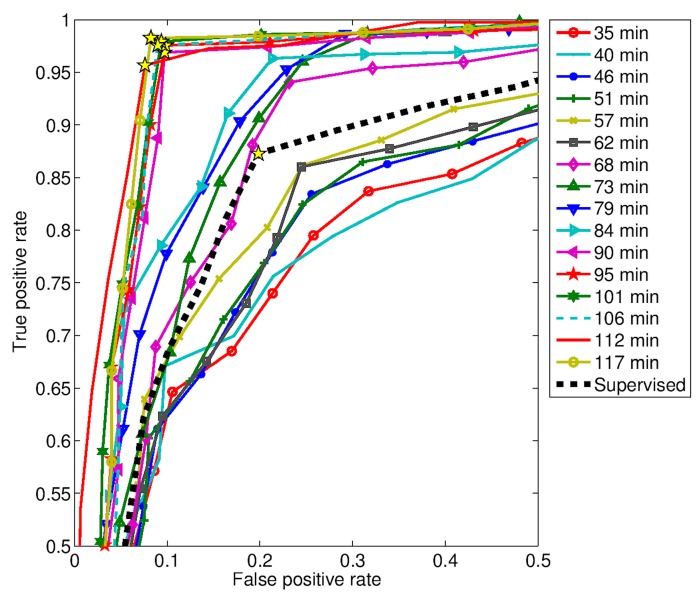
Receiver Operating Characteristic (ROC) analysis of the two detection approaches, unsupervised (for different training durations) and supervises.

**Figure 15:**
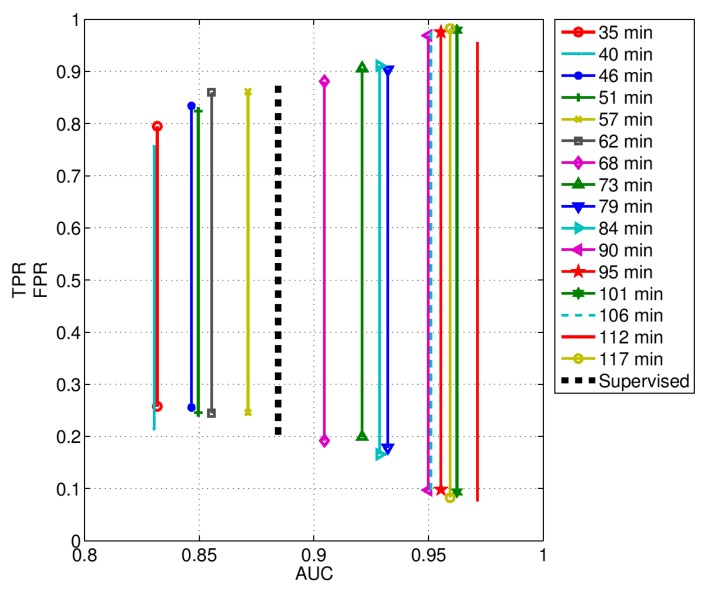
True positive rate (TPR) and false positive rate (FPR) with respect the area under the curve (AUC) for both approaches, supervised and unsupervised. The latter approach is given for various durations.

The detection performance achieved with both approaches are summarized in Table 3, considering different training duration in the unsupervised case. The experimental data were evaluated using ROC analysis in order to accommodate various computational parameters. In the unsupervised case, a ROC curve was produced at each training duration, as displayed in Figure 14 starting from a duration of 35 min. A simplified representation of detection performance for both approaches, supervised and unsupervised, is given in Figure 15. Here, it is simpler to note the performance improvement by increasing the unsupervised calibration duration.

## 5. Discussion

In general, the quality of both cardiac and respiratory signals detected by the RSS resulted quite good in comparison with the corresponding ground-truth signals, as illustrated in Figure 9 and Figure 10 respectively. However, the cardiac detection was more sensitive to movements than the respiratory one (especially to chest movements), resulting detectable only up to 3 m from the RSS. Beyond this limit, the EMD-based signal extraction strategy was not able to restore the SNR loss at the necessary level to separate the cardiac signal from the much stronger respiratory one. On the other hand, the respiratory signal resulted detectable with good accuracy up to 5 m from the sensor.

As mentioned above, the accuracy of the RSS to detect vital signs was evaluated in correspondence to some ADLs involving the three basic postures standing, sitting and lying down. In particular, the ADLs participants performed whilst in standing posture were cooking, preparing meals, and washing dishes, referred simply as “cooking” for short. The ADLs related to the sitting posture were eating at the kitchen table (referred simply as “eating”), and sitting on the couch watching TV (referred simply as “watching TV”). Regarding the lying-down posture, it was taken either during sleeping/resting or during the post-fall phase.

In both RR and HR cases, the best accuracy was achieved in correspondence of ADLs/postures without too much movements, such as, sleeping/resting, post fall, and watching TV. This explains the poor performance observed during the cooking (standing posture) activity in comparison to the other ADLs. The same applied, although at a lesser extent, in the case of the eating activity, due to some occurrence of chest oscillations. Some differences were found also in dependence of the monitored subject’s orientation. Especially in the case of HR, the most favorable orientation was toward the sensor. The subject’s position with respect to the radar antenna FoV (of about 100°) was also relevant, since the detection accuracy decreased as the subject moved away from the radial direction.

When more people were present in the RSS FoV (in addition to the monitored subject), the movement compensation strategy was robust enough as long as the distance between the monitored subject (i.e., the person closer to the RSS) and the other people was greater than 0.5 m. In such conditions, the average losses in accuracy of about 2.61% and 4.88% were observed for HR and RR, respectively, within the same distance ranges as before. The same data collection was used to evaluate the RSS performance in detecting body’s movements. To this end, micro-motion signatures and distances detected by the RSS during each validation sequence were compared with ground-truth data provided by the TOF camera. The micro-motion signatures could characterize well body’s movements in relation with performed actions, as shown in Figure 13 where a portion of sequence including a fall event is reported. As one can notice, the fall event occurred around the time sample T = 16,000 can be clearly distinguished from the previous walking actions. After the fall event, there was a period during which the subject remained unmoving until the time sample T = 24,000 when the subject recovered from the fall. Further evidence about the effectiveness of micro-motion signatures in describing body’s movements was obtained from the evaluation of the fall detection performance.

The achieved performance was quite different for the two approaches, supervised and unsupervised. More specifically, the unsupervised performance was dependent on the duration of the training phase based on “usual” ADLs. Roughly speaking, the longer lasted the unsupervised training, the higher was the detection performance.

In particular, the performance of the unsupervised approach overcame that of the supervised one, when the duration of the unsupervised training was greater than 68 min.

As one can notice, the unsupervised performance can be grouped into three groups (Figure 14 and Figure 15). The first group includes the curves from 35 min to 57 min, the second one from 68 min to 84 min, and the third one from 90 min to 117 min. The curve related to the supervised approach is placed in between the first and the second groups. From these results, hence, the following considerations can be drawn. The micro-motion signatures provided by the RSS are enough discriminative features suitable for event detection. However, their discriminative power can be improved at the cost of a greater inter-subject variability, as was done for example with the unsupervised learning approach.

## 6. Conclusions

The aim of this study was to develop and validate a RSS based on UWB-IR sensing, suitable for AAL applications. For this purpose, a comprehensive algorithmic framework for detection of both cardiorespiratory and body movements was presented and the related experimental results reported. The presented RSS was realistically evaluated by considering the detection of vital signs during the execution of various ADLs and also in presence of more than one moving subjects. Moreover, such detection capabilities were also evaluated for detecting falls and the fallen subject’s vital signs during the post-fall phase. To this end, 30 healthy volunteers divided into two aged groups were involved by simulating both ADLs and falls events, at different distances, orientations and positions with respect the RSS. The achieved results show that vital signs can be reliably detected during some ADLs and during the post-fall phase, although with accuracy varying greatly depending on the level of movements and involved body parts. The radar returns caused by movements of other people nearby were effectively compensated without significant loss of accuracy.

Furthermore, the experimental results also show the suitability of the RSS micro-movement signatures for fall detection, showing in particular the inter-subject variability which leaves room to user-customization approaches based on unsupervised learning. In conclusion, the original contribution of this work is twofold. Firstly, the promising UWB technology has been exploited for both fall detection and in-home unobtrusive vital signs monitoring. To the best of the authors’ knowledge, this is the first study that demonstrated the feasibility of detecting falls and vital signs together, using micro-Doppler spectrograms through UWB radar sensing. Secondly, the ability of the suggested micro-motion signature to effectively discriminate between ADLs and falls has been demonstrated by means of an unsupervised detection, additionally allowing to deal with the problem of the lack of fall data for training. To the best of the authors’ knowledge, in the literature, few studies attempted to do so, but only using wearable or acoustic sensors. The ongoing work is focused on further investigating the presented RSS in multi-sensor and multi-target real-life scenarios (e.g., community dwelling of older people) for simultaneous detection of vital signs and critical events.

## Figures and Tables

**Table 1:** MRM parameters adopted in this study.

$\tilde{R1}$(m)	$\tilde{R2}$(m)	R1 (m)	R2 (m)	T1 (ns)	T2 (ns)	N	$t_{s}$(μs)	$t_{i}$(μs)
0.5	1.5	0.5	1.38	13.334	19.193	96	3244.03	16,755.97
0.5	1.5	0.5	2.26	13.334	25.053	192	6488.06	13,511.94
0.5	2.5	0.5	3.13	13.334	30.912	288	9732.10	10,267.90
0.5	3.5	0.5	4.01	13.334	36.771	384	12,976.13	7023.87
0.5	4.5	0.5	4.89	13.334	42.631	480	16,220.16	3779.84
0.5	5.5	0.5	5.77	13.334	48.490	576	19,464.19	535.81

**Table 2:** HR and RR average accuracy achieved during ADLs and post-fall phase.

Activity	HR Accuracy (%)	RR Accuracy (%)
Lying down: post fall	89	93
Lying down: sleeping/resting	91	95
Sitting: eating	80	86
Sitting: watching TV	84	91
Standing: cooking	74	83
Average value	84	90

**Table 3:** Detection performance of both unsupervised and supervised approaches.

Approach	Training (min.)	Sensitivity (%)	Specificity (%)
Unsupervised	35	79.49	74.23
	40	75.61	78.56
	46	83.42	74.44
	51	82.41	75.39
	57	86.14	75.41
	62	86.01	75.53
	68	88.10	80.79
	73	90.63	80.1
	79	90.34	82.16
	84	91.10	83.44
	90	96.89	90.28
	95	97.56	90.16
	101	97.91	90.63
	106	97.57	91.02
	112	95.66	92.39
	117	98.26	91.75
Supervised	N.A.	87.27	80.15
